# *In Silico* Ventilation Within the Dose-Volume is Predictive of Lung Function Post-radiation Therapy in Patients with Lung Cancer

**DOI:** 10.1007/s10439-020-02697-5

**Published:** 2020-11-30

**Authors:** Yu Dong, H. Kumar, M. Tawhai, C. Veiga, A. Szmul, D. Landau, J. McClelland, L. Lao, K. S. Burrowes

**Affiliations:** 1 Department of Chemical and Materials Engineering, University of Auckland, Auckland, New Zealand; 2 Auckland Bioengineering Institute, Level 6, 70 Symonds Street, Auckland, 1010 New Zealand; 3 Centre for Medical Image Computing, Department of Medical Physics & Biomedical Engineering, University College London, London, UK; 4 Department of Oncology, University College London Hospital, London, UK; 5 Auckland District Health Board, Auckland, New Zealand

**Keywords:** Radiation-induced lung damage, Ventilation, Simulation, Computer model, Respiratory, Cancer

## Abstract

Lung cancer is a leading cause of death worldwide. Radiation therapy (RT) is one method to treat this disease. A common side effect of RT for lung cancer is radiation-induced lung damage (RILD) which leads to loss of lung function. RILD often compounds pre-existing smoking-related regional lung function impairment. It is difficult to predict patient outcomes due to large variability in individual response to RT. In this study, the capability of image-based modelling of regional ventilation in lung cancer patients to predict lung function post-RT was investigated. Twenty-five patient-based models were created using CT images to define the airway geometry, size and location of tumour, and distribution of emphysema. Simulated ventilation within the 20 Gy isodose volume showed a statistically significant negative correlation with the change in forced expiratory volume in 1 s 12-months post-RT (*p *= 0.001, *R* = − 0.61). Patients with higher simulated ventilation within the 20 Gy isodose volume had a greater loss in lung function post-RT and vice versa. This relationship was only evident with the combined impact of tumour and emphysema, with the location of the emphysema relative to the dose-volume being important. Our results suggest that model-based ventilation measures can be used in the prediction of patient lung function post-RT.

**Electronic supplementary material:**

The online version of this article (doi:10.1007/s10439-020-02697-5) contains supplementary material, which is available to authorized users.

## Introduction

Lung cancer is the most commonly diagnosed cancer and is the leading cause of cancer death worldwide.^5^ Radiation therapy (RT) is an important component of the cure or palliation of patients with lung cancer. RT uses ionising radiation to destroy or damage cancer cells, however normal lung tissue is also damaged which can lead to radiation-induced lung damage (RILD), including radiation pneumonitis and radiation fibrosis. RILD is a dose-limiting factor in chest RT and typically results in a loss in lung function. One study has shown mean reductions in forced expired volume in 1 second (FEV_1_) and diffusion capacity for carbon monoxide (*D*_LCO_) as high as 24.2% and 20.1%, respectively, in patients 12-months post-RT receiving concurrent chemoradiotherapy.^25^ In the majority of lung cancer patients—about 90% of whom have a history of smoking—RILD compounds pre-existing smoking-related regional lung function impairment.^29^ Because of the risk of decreasing function with RT in an already compromised lung, the RT dose for lung cancer is often limited to subtherapeutic doses or a patient may even be denied treatment based on estimated risks of RILD. However, in some cases, lung function is preserved or can even improve post-RT.^18^ This could be due to the reduction or removal of a tumour that was previously obstructing major airways. Another hypothesis is that RT can reduce hyperinflation of pathological tissue within the dose-volume in patients with emphysema, hence improving elastic recoil and function in the surrounding tissue.^3^ It is currently not possible to use clinical or image-based measures to understand these contrasting mechanisms or accurately identify the impact that RT will have on a given patient’s lung function.

The complexity of predicting RT toxicity risk relates to the large variation in individual response to RT, with one study showing a 20-fold difference in radiosensitivity.^9^ Previous modelling studies have typically applied statistical modelling techniques (univariate or multivariate), machine learning, or predictive modelling methods^37^ to establish patient-based biomarkers to identify which patients are at higher (or lower) risk for RILD. These studies have included analysis of dosimetry parameters (for example mean lung dose or V20, the volume of tissue irradiated with > 20 Gy),^20^ regional perfusion (Q) and/or ventilation (V),^2^,^12^ pre-treatment computed tomography (CT) characteristics,^10^ patient genetics,^38^ or molecular biomarkers.^20^ None have been able to predict post-RT loss in lung function for patients on a personalised level, because often only one of these variables is considered but in reality, various aspects are at play. There are no accurate models or metrics that can currently predict patient outcomes post-RT, therefore clinical decision-making for an individual is currently a ‘best-guess’ based mainly on dosimetry parameters, patient age and prognosis, and global pre-treatment lung function (measured *via* standard pulmonary function tests, PFTs). If more accurate predictions of lung damage and the resultant change in lung function were possible, more patients could potentially be treated with RT and/or the dose could be increased with consequential therapeutic gain.^13^,^33^

Although most patients will present with heterogeneous lung function resulting from the tumour and any comorbidities, current RT planning and decision making do not account for this. A previous study has shown that four-dimensional computer tomography (4DCT)-derived regional ventilation within the irradiated lung volume is predictive of patient lung function after stereotactic ablative radiotherapy (SABR).^2^ However, obtaining 4DCT-based ventilation measurements requires specialised post-processing software and is time-consuming, of low quality, or in many cases is not possible. In addition, imaging measurements, like 4DCT, are unable to explain the mechanisms behind such predictive biomarkers. Previously, we have developed an *in silico* modelling platform (consisting of several different models that can be loosely coupled together) to create patient-based lung models capable of simulating regional lung function, for example.^8^,^30^,^31^ We hypothesised that by simulating patient-based regional ventilation—including the impact of the tumour and any coexisting emphysema, combining the impact of the dose map, regional ventilation pre-RT based on CT characteristics—we could improve predictions of the impact of RT on lung function and increase our understanding of the important factors or mechanisms leading to variation in patient outcomes. To test this hypothesis, 25 patient-based models were created using CT images pre-RT. The patient-based tumour and any coexisting emphysema were included in the model. Patient-based *in silico* measures of ventilation (pre-RT) were compared with spirometry measurements of patient’s lung function pre- and post-RT.

## Materials and Methods

### Patient Data

Our study included 25 patients treated with conventional chemoradiotherapy. These patients were a subset of patients enrolled in the IDEAL-CRT (Isotoxic Dose-Escalated Radiation Therapy and Concurrent Chemotherapy) clinical trial, a phase 1/2 multicentre trialrun across eight centres in the United Kingdom. IDEAL-CRT collected longitudinal data on 120 stage II/III non-small-cell lung cancer (NSCLC) patients,^18^ before and after-RT (3, 6, 12 and 24 months post). The study was run following the Declaration of Helsinki and with the approval of all relevant ethical bodies and regulatory authorities, and the use of a subset of this data was approved by the University of Auckland Human Participants Ethics Committee (UAHPEC), reference 020572. The subset of 25 patients was selected based on the following criteria: (i) baseline (pre-RT) and 12 month follow up patient data was available, (ii) all dosimetry and RT dose information was available, and (iii) patients were all treated with 6-week fractionation. The data used here included forced expiratory volume in one second (FEV_1_), forced vital capacity (FVC) and diffusion capacity to carbon monoxide (D_LCO_) in patients pre-treatment (baseline) and 12-months post-treatment and CT scans (median resolution: 0.82 × 0.82 × 2.5 mm, range across centres: 0.64 × 0.64 × 0.80 – 0.98 × 0.98 × 5.0 mm) at baseline (deep inhalation breath-hold) for 25 lung cancer patients. A summary of patient information for the 25 subjects is shown in Table 1.

**Table 1:** Mean (± standard deviation, SD) patient demographics, tumour volume, and lung function information.

Age, (years)	66.7 (± 9.6)
Sex: male/female	20/5
Height, (m)	1.7 (± 0.1)
Weight, (kg)	81.4 (± 16.2)
BMI	29.2 (± 5.0)
Tumour location, (central/peripheral)	23/2
Tumour volume, (cm^3^)	107.3 (range 14–317)
FEV_1_ (L)	2.1 (± 0.5)
FEV_1_ (% pred)	74.3 (± 23.6)

*FEV*_*1*_, forced expiratory volume in one second; *% pred* is % predicted according to European Respiratory Society (ERS) 1993 and the Third National Health and Nutrition Examination Survey (NHANES III) population standards^14^,^28^

**Figure 1:**
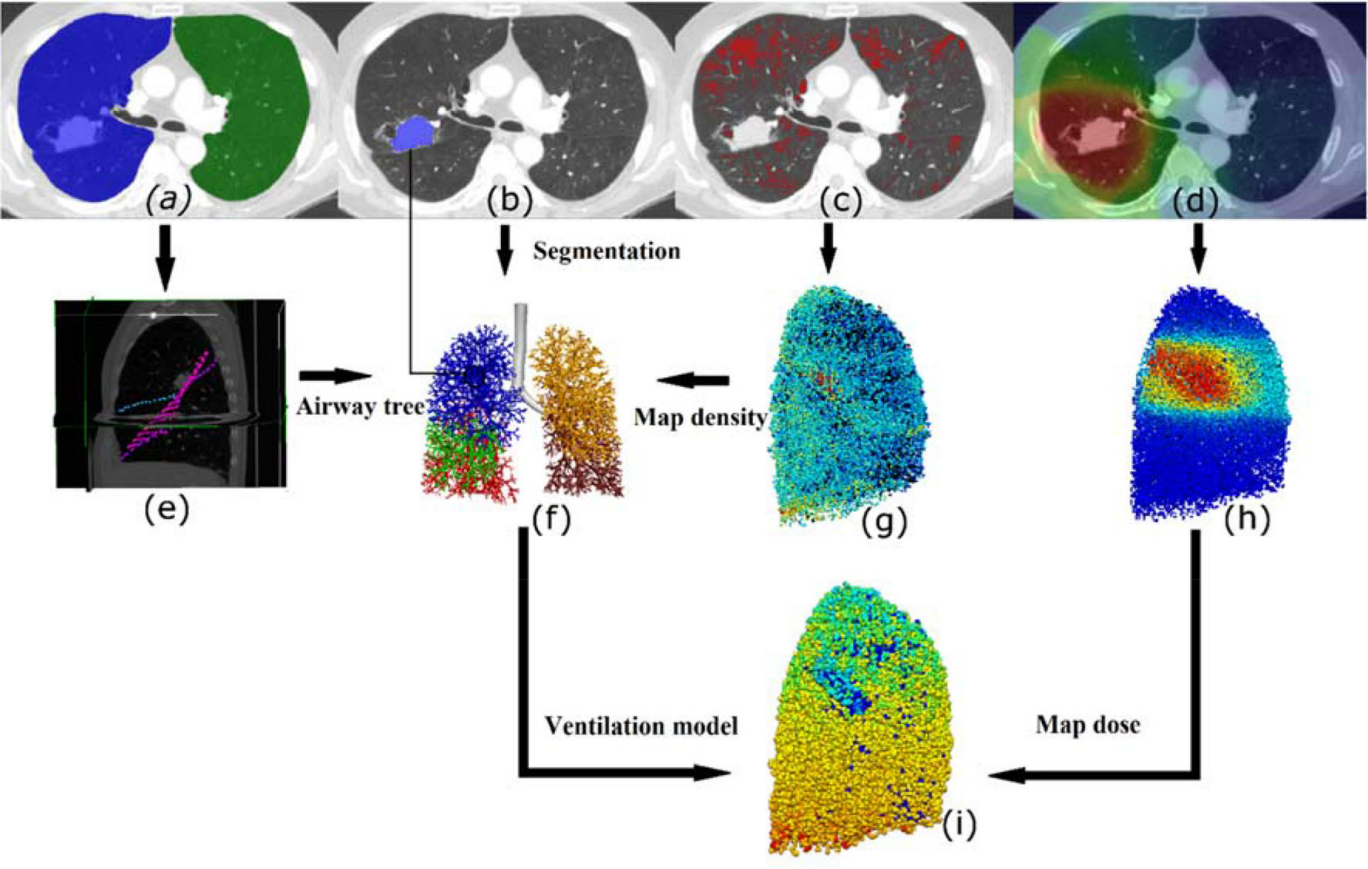
Illustration of the workflow for creating patient-based models. (a-d)Segmentation of patient CT scans provided: (a) left and right lung volumes, (b) gross tumour volume (GTV), and (c) emphysematous tissue. The dose distribution (d) was overlaid on the CT images. (e) Fissures were manually extracted, and (f) airway trees were grown into patient-based lobar volumes. The GTV was mapped onto the model and airways within the GTV were partially constricted. (g) Emphysema and (h) dose (colour spectrum indicates dose ranging from 0 Gy dark blue to 20 Gy light blue, and 65 Gy red) were mapped from CT onto the airway model. (i): Side view of one patient-based (left lung) model indicating normalised ventilation solution (ventilation/mean ventilation in the whole lung) including constriction due to patient’s tumour (colour indicates normalised ventilation ranging from 0 dark blue to 1.5 red).

An overview of the methods used in this study is presented in Fig. 1. In brief, we use patient volumetric CT data to create personalised models, including representation of the lung shape, lobes, central airways, tumour volume, and distribution of emphysema. Figures 1a–1d illustrates the information acquired from CT for one subject, including (a) definition of the left (green) and right (blue) lungs and lobe fissures, (b) tumour (blue) location, and (c) emphysematous tissue (red). The fissures were identified manually (Fig. 1e) and airway trees were generated into the patient-based lobe volumes. The tumour volume was mapped onto the airway model and airways within the tumour volume were partially constricted (Fig. 1f). Thresholding was used to identify regions with emphysema, and this was mapped onto the patient-based airway model (Fig. 1g). Regional ventilation was predicted after including patient-based dysfunction (Fig. 1i) and the 3D dose map (Fig. 1h) was overlaid onto the model for subsequent analysis.

### CT-Based Measures: Segmentation and Emphysema Quantification

#### Segmentation

The geometry of the left and right lungs and central airways were automatically segmented using the open-source software Pulmonary Toolkit (PTK), available at https://github.com/tomdoel/pulmonarytoolkit/wiki/Pulmonary-Toolkit). Airways were converted to a centreline approximation (1D lines, plus radius definition) from the segmented airway lumens using a skeletonisation procedure. The methods applied in this software have been described previously in Burrowes *et al*.^7^ Lobar fissures for both left and right lung were segmented manually using in-house software. Patient-specific tumour boundaries were manually segmented for each subject to provide the gross tumour volume (GTV) outline.

#### Emphysema Quantification

Regions of emphysema manifest as lower density units; emphysema is typically quantified by applying an intensity threshold to a density mask from a maximum inspiratory CT scan. To create the density mask, voxels containing large airways, vessels, and lung surface were excluded automatically by the PTK software, using intensity thresholding, allowing classification of the parenchymal tissue density. The most commonly used threshold for emphysema quantification is − 950 HU, such that all voxels with a HU value less than − 950 HU are considered to contain tissue that is emphysematous.^19^ However, tissue density, or HU, is highly dependent on imaged lung volume.^21^ Due to intra-patient variability in the inhalation level in the patient data, the emphysema threshold value was scaled for each patient based on the inhalation level of the subject. To do this, the standard equation relating density (${\rho}$, in g cm^−3^) and HU (Eq. 1) was rearranged and the assumption of constant mass at varying lung volumes was applied. The inhalation level was represented by the ratio of the volume of air measured from the CT scan using PTK ($V_{\text{air}}$) and the predicted total lung capacity ($V_{\text{TLC}}$). $V_{\text{TLC}}$ was calculated using the ERS 1993 reference equation.^28^ Patient-based TLC measurements were not available in this study. The scaled emphysema threshold value (${\text{HU}}^{*}$) was calculated using Eq. 2.

1 $$ \rho = \frac{\text{HU}}{1000} + 1 $$

2 $$ HU^{*} = \left( {\left( {\frac{ - 950}{1000} + 1} \right)\left( {V_{\text{TLC}} /V_{\text{air}} } \right) - 1} \right) \times 1000 $$

Any regions that were classified as emphysema from the imaging, were incorporated into the modelling framework using different local compliance to incorporate the impact of emphysema damage on ventilation function (see “Ventilation Model” section).

### Geometric Model

#### Airway Tree Generation

Patient-based conducting airways were generated using a combination of imaging data (central airways and lobe shape) and a computational algorithm that produced additional conducting airways, to the level of the acinus, into the lung volume. This algorithm produces a volume-filling branching network that is morphometrically accurate and has been published and applied previously.^4^,^31^ Further details can be found in the Supplementary Material.

Patient-specific CT tissue density (extracted from CT using Eq. 1) was mapped onto each model acinus and was used to determine the coefficient of variation of density across all model acini for each patient. The voxel size of the CT scans was typically smaller than the actual size of an acinar unit, therefore the mean density of a 125 mm^3^ (5 mm × 5 mm × 5 mm) cube from CT was used to represent the acinar unit density for each acinus within each model.

### Ventilation Model

An existing model of ventilation, developed by Swan *et al*.,^30^ which simulates quiet breathing, was applied in this study. A summary of the model components is given here, with additional details in the Supplementary Material. This model provides a prediction of the time-average ventilation within each airway and the acinar unit in the lung network. All simulations were conducted in the upright posture to align with the PFT measurements which are obtained in the upright position. A sinusoidal, time-dependent, pleural pressure (*P*_pl_) gradient (5 s for both inspiration and expiration, mean $P_{\text{pl}}$ ranges from − 5.0 cmH_2_O at functional residual capacity (FRC) to − 8.2 cmH_2_O at end of inspiration before disease added, $P_{\text{pl}}$ varies based on patient-specific target volume and diseases) was applied at each acinus and equations were solved to predict flow at each time point during a breath. $P_{\text{pl}}$ was modified during the simulation to ensure that the volume of inspiration was equal to the predicted tidal volume for each subject. Baseline FRC and tidal volumes for each patient model were estimated using height, weight, gender, and age for a given patient.^28^ These estimations were then used as boundary conditions for the simulation of ventilation (mean FRC across all subjects was 2.43 ± 0.40 l, mean tidal volume 0.35 ± 0.07 l). Tidal volume was assumed to be constant within each subject before and after the disease effects were applied due to a lack of data describing whether this changes. The initial volume of each acinus at FRC was scaled randomly based on the coefficient of variation of measured density for each patient (with tumour, emphysema, and vessels excluded), and a linear gradient (based on the ratio of maximum and minimum density values to the mean density value for each subject—derived from CT data) was applied to change the acinus volume at FRC along the gravitational axis (upright in this case). The model of Swan *et al*.^30^ was modified to include: (1) the impact of the tumour and (2) the impact of emphysema.

#### Impact of the tumour

The presence of the tumour was incorporated into the model in two ways: stiffening of terminal units and airway constriction. Acinar units located within the GTV were identified and assigned to have increased stiffness meaning they had little to no expansion during ventilation from FRC to tidal volume. To achieve this, the strain energy density coefficient $\xi$ was set to be 15000 Pa for all terminal units within the GTV, so that the normal healthy tissue was six times more compliant than the tissue within the tumour volume. The value of $\xi$ for tumour tissue was chosen based on the pressure-volume curve for fibrotic lung tissue reported by Pride *et al*.^27^

Tumours that develop in the lungs may fully or partially occlude the surrounding airways. Over 50% of advanced-stage lung cancer patients have narrowed central airways, and terminal airways can be blocked by the tumour as well.^35^ To incorporate this effect in our ventilation model, patient-based tumour volumes were mapped onto each model and any airways within the GTV were constricted. Proximal airways (radius > 2 mm)^22^ were constricted to 50% of their ‘normal’ radius value and distal airways were constricted to 30% of their initial, normal radius value to represent an intermediate amount of occlusion.

#### Impact of Emphysema

For any acini that were classified as emphysema, according to the scaled HU threshold for that subject (Eq. 2), the CT-based tissue density was incorporated into the boundary conditions of the model such that emphysema tissue was hyperinflated and therefore had reduced compliance. In this study, the isotropic stretch ratio (this is the stretch from the undeformed reference volume to FRC assuming isotropic stretch) $\lambda$ was set to be a function of density in those regions determined to be emphysema as follows:

3 $$ \lambda = \frac{{k^{1/3} }}{{\rho^{1/3} }} + 1.15, $$

where ${\rho}$ (g cm^−3^) is the density measured from CT, $k = 1\;{\text{g}}\,{\text{cm}}^{ - 3}$. In this method, regions with emphysema and decreased density have consequential increases in the isotropic stretch ratio ${{\lambda }}$ which leads to a decrease in compliance.

### Application of the Model

#### Can Simulated Ventilation be Used as a Predictor of Lung Function Post-RT?

The patient-specific 3D RT dose map was overlaid onto the model to enable calculation of the amount of ventilation within the dose region. To enable this, registration from the RT planning CT scan to the baseline diagnostic CT image (from which the model was created) was done for all subjects using the open-source software package NiftyReg (sourceforge.net/projects/niftyreg). NiftyReg uses the B-Spline Free-Form Deformation algorithm.^23^ The registrations used a multiresolution approach, local normalised correlation coefficient (LNCC) as similarity measure (Gaussian kernel standard deviation is 5), velocity field integration to generate the deformation and bending energy as the regularisation term.

The association between simulated ventilation within different isodose volumes and the change in lung function 12-months post-RT was investigated. All acinar units receiving > 20 Gy and > 30 Gy were identified in each patient-based model. The mean ventilation in all acini within these isodose volumes, divided by the mean ventilation for the whole lung was calculated for each subject and is referred to as V_R20_ and V_R30_, respectively. T-test was performed to assess if there was any statistically significant correlation (*p* ≤ 0.05) between the change in lung function, using FEV_1_ and D_LCO_, 12-months post-RT and V_R20_ and/or V_R30_.

#### Simulation, Validation, and Sensitivity Analysis

Simulations were performed in the upright posture before and after disease effects were added. Baseline patient data and model-based simulated data were correlated with the change in lung function post-RT to see which variable(s) would provide the best predictor of outcomes. The coefficient of variation (CoV) of simulated ventilation within each patient-based model was plotted as a function of measured FEV1 (% predicted). Simulated values were compared against the same measures derived from 4D-CT imaging to confirm the consistency of the model with previously measured outcomes.

To improve the explanation of our findings, a single patient-based model was used to perform a sensitivity analysis of the effect of adding emphysema into the model. All patient-based variables were included and conserved except for the emphysema which was artificially included in the model to test the impact of the distribution of emphysema and the importance of its proximity to the tumour. The patient-based model used was derived for a 68-year-old male with a tumour size of 123 cm^3^, FEV_1_: 1.96 L, FEV_1_ % predicted: 70.3%, *D*_LCO_: 20.1, *D*_LCO_ % predicted: 82.0%, FVC: 2.36 L, FVC % predicted: 64.1%, V20: 23.1%, adjusted emphysema threshold: 0.069 g cm^−3^. The proportion of emphysema was incremented by setting the density of every *n*th ($n = 100/\%  {\text{emphysema}}$, for example when considering 10% emphysema every 10th acinus was altered) acinar unit to be half of the emphysema threshold, thereby inducing the emphysema impact in the model. The distribution of emphysema was controlled by selectively including the emphysema either inside or outside of the V20. When assessing the impact of emphysema inside V20, all emphysema outside V20 was excluded and vice versa. The impact of the proximity of the emphysema to the tumour was studied by selectively excluding or including emphysema in the V10 isodose volume. In this way, we aimed to analyse the impact of the distribution of the emphysema in relation to the RT treatment volume.

#### Statistical Analysis

Linear correlation analysis was performed to assess whether relationships existed between any of the clinical variables (at baseline, pre-RT treatment) or model simulated variables (V_R20_, V_R30_, and CoV of ventilation) and the change in lung function 12-months post-RT (including FEV_1_, FEV_1_ % predicted, FVC, FVC % predicted, *D*_LCO_, *D*_LCO_ % predicted, and the ratio of FEV_1_/FVC). Pearson correlation coefficients (*R*) and uncorrected *p*-values are presented.

## Results

**Figure 2:**
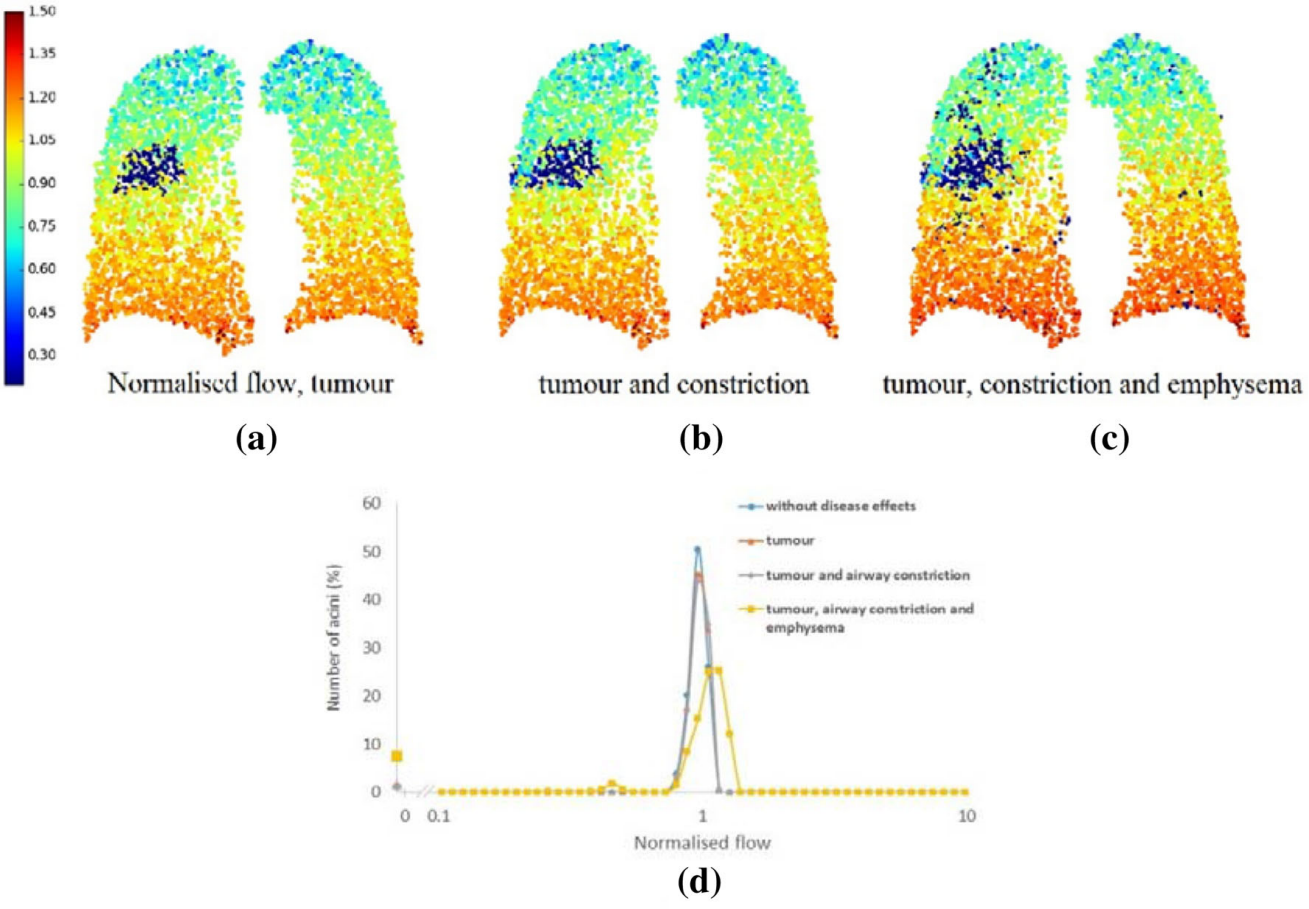
Changes in ventilation solution in a single patient-based model due to the impact of tumour and emphysema by applying the following impacts: (a) increased acinar stiffness (meaning the lung tissue cannot inflate in the tumour region); (b) increased acinar stiffness and airway constriction; (c) increased acinar stiffness, airway constrictions, and reduced acinar compliance (this reduced compliance mimics the hyperinflated emphysema regions meaning they are harder to inflate). Figures (a) to (c) show 2D cross-section of a coronal slice through the model with the colour spectrum indicating normalised flow values. (d) Shows a histogram plot for the predicted ventilation in acinar units without disease effects and with increased acinar stiffness, increased acinar stiffness and airway constrictions, and three effects combined (increased acinar stiffness, airway constrictions, and reduced acinar compliance). This patient had a tumour volume = 173 cm^3^, baseline FEV_1_ % predicted = 75%.

The mean normalised ventilation distribution was similar across all subject models before disease effects being added, however, there was some variation across the subjects due to differences in the airway network geometry, lung sizes, and the initial volume of acini within the patient-based models. Figure 2 shows the ventilation solution through a 2D cross-sectional slice from a single patient-based lung model illustrating the addition of the disease components. A colour spectrum is used to demonstrate the ventilation values within each model acinus. A gravitational gradient is evident with increased ventilation in the gravitationally-dependent (caudal) region. Figure 2a illustrates the effect of (acinar) tissue stiffening in the GTV; the acinar tissue within the tumour volume does not expand or receive any airflow.

When constriction of airways within the GTV was added to the model the volume of lung tissue impacted was larger because ventilation to all acinar units distal to the constricted airways within the GTV was impacted by the airway constriction, Fig. 2b. Figure 2c shows the ventilation distribution after the addition of emphysema into the model alongside the tumour effects. Tissue regions that were classified as emphysematous received lower ventilation than normal acinar units. A histogram of the distribution of ventilation across all acinar units in a single patient-based lung model is shown in Fig. 2d; results are indicated for each stage of the model from no disease to all disease effects. Without disease, a normal ventilation histogram is evident, with a single ventilation peak around 1.0 (normalised). The addition of the tumour effects showed a modest impact on the ventilation distribution with the largest change being observed when emphysema was added alongside the tumour effects. With all disease effects included, the proportion of alveoli receiving no ventilation in this single patient-based model increased to nearly 10% of the total ventilation and the distribution became broader with decreased ventilation in a noticeable proportion of the acinar tissue. The peak of the distribution move to larger flows due to healthier tissue regions receiving increased flow to compensate for the dysfunctional tissue.

**Figure 3:**
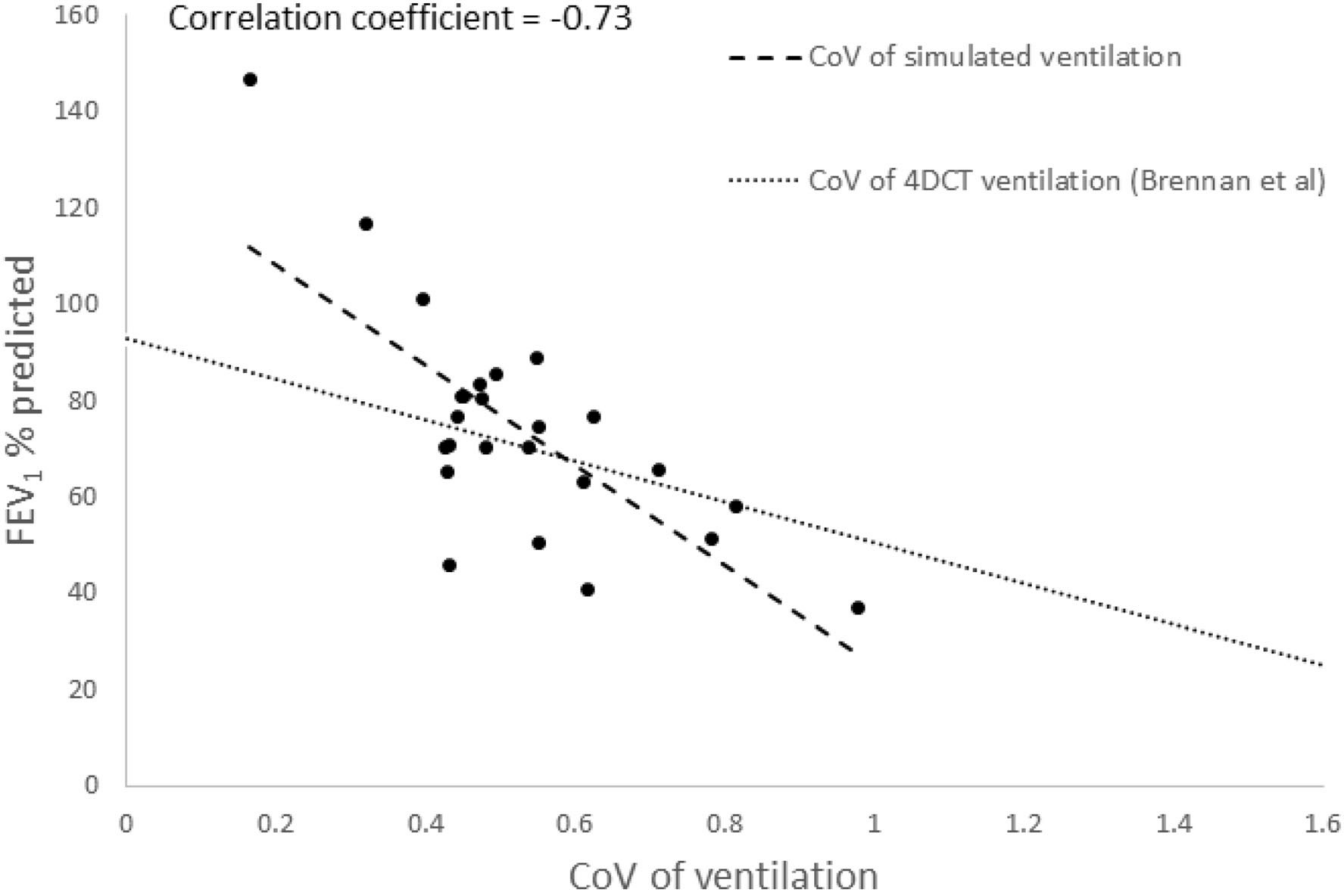
The relationship between FEV_1_ % predicted and the coefficient of variation (CoV) of simulated ventilation in the patient-based lung models for all 25 lung cancer patients (Pearson correlation coefficient, *R* = − 0.73, *p* = 0.00005). The simulated data were compared with clinical data from a study using 4DCT to estimate ventilation in 98 lung cancer patients by Brennan *et al*.^6^

The ability of the model to represent ventilation in lung cancer patients was validated by comparing metrics from the simulated ventilation distribution against FEV_1_ measurements for all subjects. A statistically significant correlation between the coefficient of variation (CoV) of simulated ventilation (*V*, using flow rates in the acini only) and measured pre-treatment FEV_1_ % predicted was found (*R* = − 0.73, *p* = 0.0005) as shown in Figure 3. This indicates that FEV_1_ (% pred) decreases with increasing heterogeneity (CoV) of simulated ventilation, indicating a lung with less efficient function. Brennan *et al*. previously showed a correlation *R* = − 0.72 (*p* < 0.01) between FEV_1_ and measured ventilation data (obtained from 4DCT) in patients with lung cancer.^6^ In the study by Brennan *et al*., 4DCT data sets and spatial registration were used to compute 4DCT-ventilation images using a density change-based and a Jacobian-based model. 4DCT images were registered to provide a map of estimated ventilation. In their cohort of 98 patients, 65% had stage II/III lung cancer, and 29% of the lung cancer patients had pre-existing chronic obstructive pulmonary disease (COPD). Our data is in good agreement with the 4DCT measurements of ventilation, providing evidence that our model realistically represents regional ventilation in lung cancer patients.

**Figure 4:**
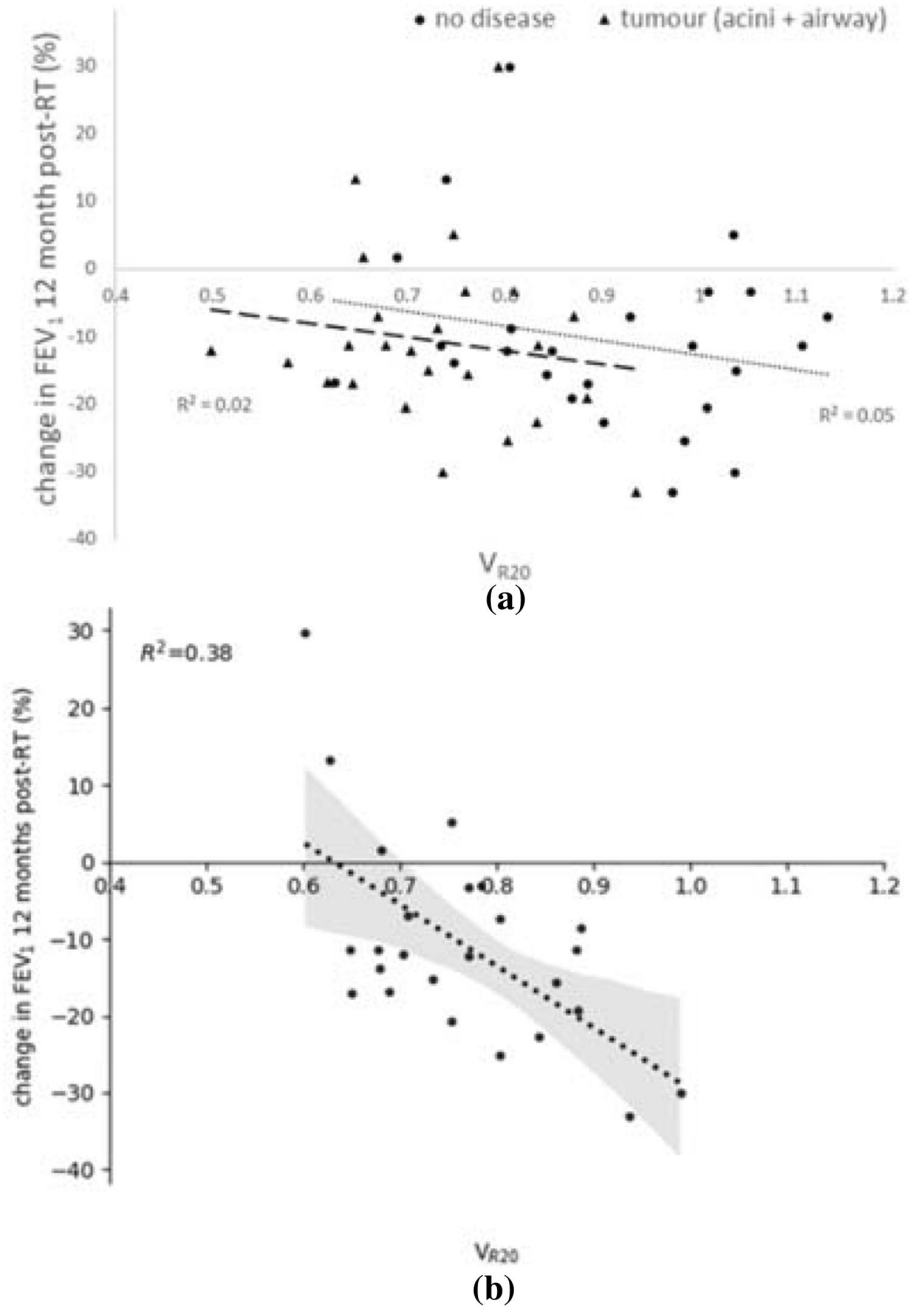
Correlation between V_R20_ (mean ventilation in the tissue receiving > 20 Gy divided by mean total lung ventilation) and the patient measurement of the percent change in FEV_1_ (L) 12-months post-RT. (a) Simulated V_R20_ with no disease and with only the tumour added to the model. (b) Simulated V_R20_ with all disease effects (tumour + emphysema) added into the simulation. The slope of the line in (b) is − 80.12 (95% CI 44.5) and the intercept is 50.6 (CI 34.3).

Table 2 presents the Pearson correlation coefficients (*R*) after performing linear regression statistical analysis. Variables that had a statistically significant correlation (*p < 0.05, **p < 0.01) are indicated. The strongest correlation was observed between V_R20_ and the change in FEV_1_ post-RT with an *R*-value of − 0.61 (*p* = 0.001), this relationship is plotted in Fig. 4b. The same statistical analysis was performed using the V30 isodose volume (including V_R30_) but no significant correlations were found and results are not shown.

**Table 2:** Pearson correlation coefficients (R) assessing the relationships between clinical, image-based and model-based variables and the measured change in lung function post-RT.

	Change in percent predicted			Percent change in absolute value			
	FEV_1_	FVC	D_LCO_	FEV_1_	FVC	D_LCO_	FEV_1_/FVC
Tumour relative size (%)	0.27	0.22	0.23	0.14	0.33	0.03	− 0.20
V20 (%)	− 0.15	− 0.23	− 0.27	− 0.15	− 0.34	− 0.05	0.16
Emphysema in whole lung (%)							
Using threshold < 950HU	0.06	0.18	− 0.19	0.14	0.16	− 0.11	0.02
Using adjusted HU threshold	0.17	0.14	− 0.16	0.26	0.16	− 0.05	0.16
Emphysema in V20 (%)							
Using threshold < 950HU	0.12	0.08	− 0.05	0.16	0.14	0.04	0.06
Using adjusted HU threshold	0.12	0.04	− 0.04	0.15	0.13	0.04	0.05
FVC	− 0.11	− 0.22	− 0.31	− 0.04	− 0.13	0.12	0.09
FVC % predicted	− 0.39	− 0.56** (*p* = 0.003)	− 0.21	− 0.21	− 0.40* (*p* = 0.046)	0.13	0.21
FEV_1_	− 0.35	− 0.28	− 0.25	− 0.41* (*p* = 0.041)	− 0.25	0.13	− 0.26
FEV_1_ % predicted	− 0.49* (*p* = 0.012)	− 0.40* (*p* = 0.05)	− 0.16	− 0.38	− 0.37	0.11	− 0.08
D_LCO_	0.11	0.19	− 0.10	− 0.03	0.08	0.10	− 0.16
D_LCO_ % predicted	− 0.05	− 0.05	− 0.11	− 0.08	− 0.10	0.16	− 0.02
FEV_1_/FVC	− 0.29	− 0.13	− 0.03	− 0.42* (*p* = 0.0036)	− 0.17	0.02	− 0.37
CoV of V	0.12	− 0.18	0.16	0.03	0.11	0.19	− 0.04
V_R20_	− 0.68** (*p* = 0.0002)	− 0.60	− 0.49* (*p* = 0.015)	− 0.61** (*p* = 0.001)	− 0.74** (*p* = 0.00004)	− 0.31	− 0.08

*V20* percent volume of lung received over 20 Gy, *HU* Hounsfield units; Emphysema % is volume of emphysema divided by volume of lung; Adjusted HU values are emphysema threshold values scaled as a function of lung volume; *FVC* forced vital capacity at baseline (pre-RT); *FEV*_*1*_ forced expiratory volume in 1 second at baseline; *D*_*LCO*_ diffusion capacity of the lung for carbon monoxide; *CoV of V* coefficient of variation of simulated acinar ventilation; *V*_*R20*_ the mean ventilation in all acini within 20 Gy dose volume, divided by the mean ventilation for the whole lung. *p < 0.05 and **p < 0.01 are statistically-significant correlations, p-values are included for those parameters with statistically-significant correlations

**Figure 5:**
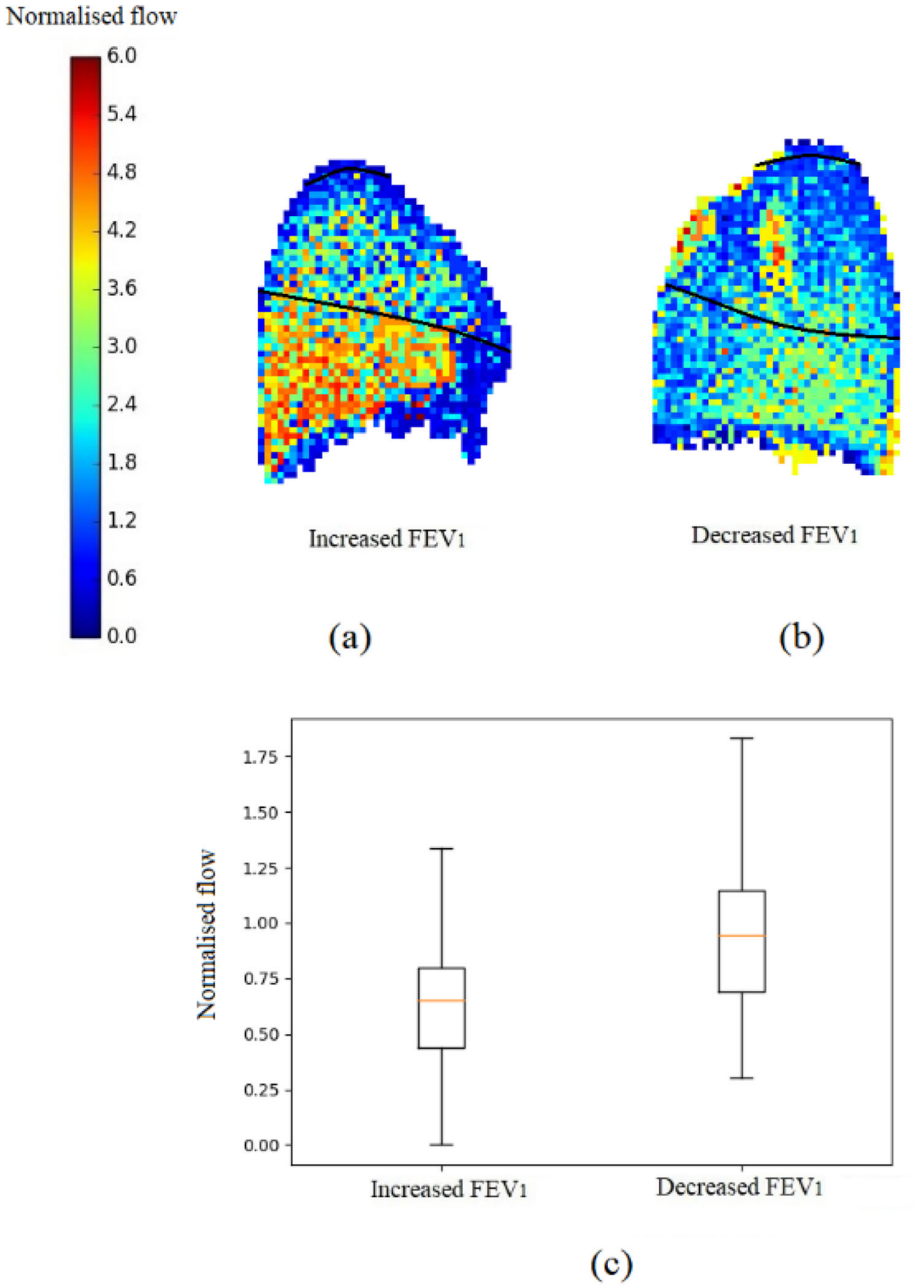
Demonstration of two patient-based models, despite a very similar V20 in each case (27 and 23% of the total lung, respectively) one patient had an increase in FEV_1_ and the other a decrease. (a, b) Display a side view of the ipsilateral lung with the sum of the normalised flow (predicted in the models) projected on the plane indicating the ventilation within the 20 Gy dose region (a: $V_{{{\text{R}}20}}$ = 0.60 and b: $V_{{{\text{R}}20}}$ = 0.99, these two subjects are outliers observed in Fig. 4b); the black lines (drawn around the upper lung) indicates the isodose area. The patient with decreased FEV_1_ had higher ventilation (V_R20_) in the irradiated region. (c) The boxplot shows the distribution of normalised flow in the 20 Gy isodose volume from each model.

The relationship between V_R20_ and the change in FEV_1_ 12-months post-RT is plotted in Fig. 4b. There was a significant correlation between these variables (*R* = − 0.61, *p* = 0.001); this relationship was only evident when both the tumour and emphysema disease effects were included together. In particular, it was observed that those patients who had an improvement in lung function post-RT had lower V_R20_ in the simulations. In contrast, those who had large decreases in lung function post-RT had a higher simulated V_R20_ (in other words, there was more function in the model in the V20 irradiated volume). Simulated V_R20_ when including only the patient-specific tumours (Fig. 4a) or simulations only including emphysema showed no relationship with the change in lung function post-RT treatment. There are four outliers observed in Fig. 4b, these are the interesting cases that this type of analysis provides the greatest benefit. Patients who have the potential to gain lung function post-RT can possibly be treated with slightly less caution (i.e. higher dose) without fear for their lung function post-RT. And those patients who have the greatest loss in lung function are the ones we want to be able to identify and work with more cautiously. These outliers are analysed in further detail in Fig. 5 to see what made their outcomes so disparate.

Figure 5 illustrates two representative patient models: one patient who had an increase in FEV_1_ post-RT (a) and one patient with a decrease in FEV_1_ post-RT (b), these were two of the extreme cases observed in Fig. 4b. These patients had a similar V20 volume ((a) 27% and (b) 23% of the patient’s total lung volume) and similar tumour volumes ((a) 115 cm^3^ and (b) 123 cm^3^). Patient (a) had a lower simulated V_R20_ (0.60) and patient (b) had a higher V_R20_ (0.99) with all disease changes included (Fig. 5c). Part of these differences was due to the anatomical location of the V20 volume ((a) ranges from 36 to 90% and (b) from 46 to 87% of lung height along the gravitational axis), this resulted in differences in V_R20_ even before disease changes were added. Before disease changes, patient (a) had a lower V_R20_ (0.8) compared to patient (b, 1.0), this is because patient (a) had a larger proportion of the V20 volume in the gravitationally-independent lung region ((a) 61% and (b) 46% of acini in V20 had flow lower than mean flow of the whole lung without disease effects). The other difference found was that patient (a) had a large proportion of their emphysema (a volume equivalent to 0.38% of total lung volume) inside V20 with a minimal amount of emphysema directly outside V20 (0.001% was located between the 20 Gy to 10 Gy isodose volumes). This distribution of emphysema caused a decrease in V_R20_. Patient (b) had some emphysema within V20 (0.62% of total lung volume in the posterior segment of the upper lobe), however, they had a larger amount of emphysema directly outside V20 (a volume equivalent to 1.01% of their total lung volume was located in between the 20 Gy and 10 Gy isodose volume) which caused an increase in V_R20_ for this patient.

**Figure 6:**
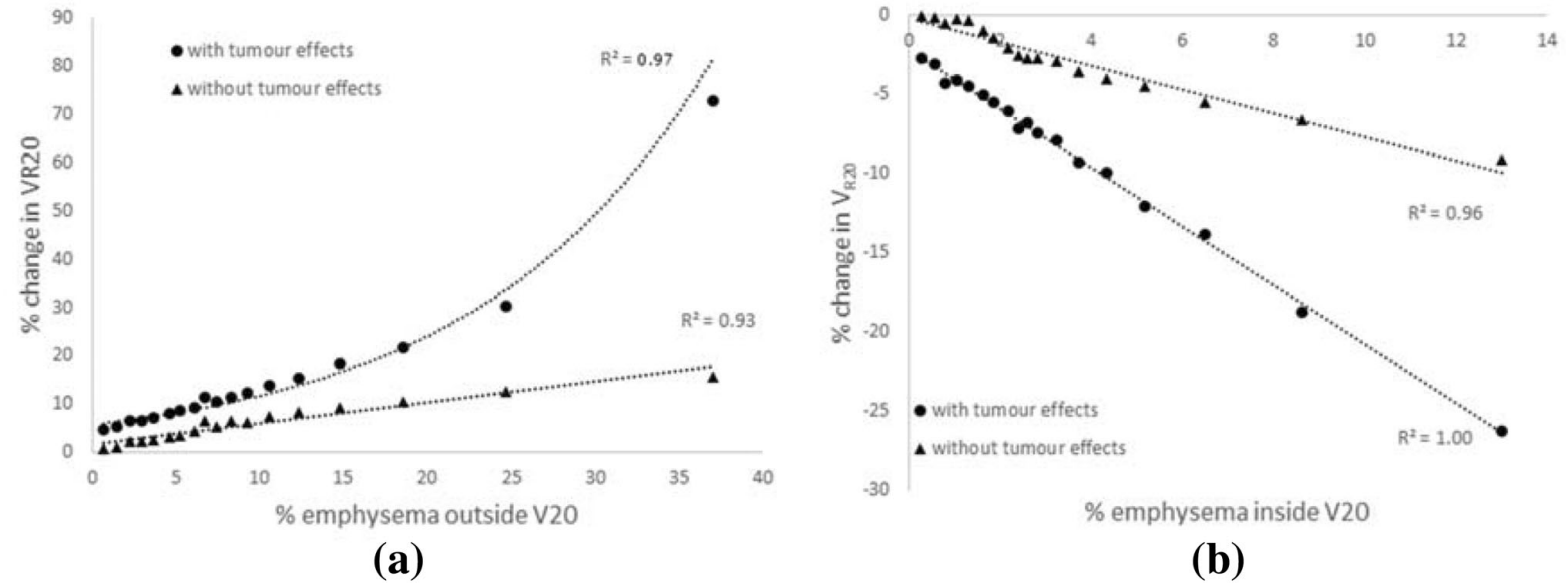
The impact of emphysema location on simulated *V*_R20_ in a single patient-based model with and without the tumour included. (a) % change in *V*_R20_ (where the change in *V*_R20_ is referenced to *V*_R20_ with 0% emphysema) is plotted as a function of the % emphysema outside V20. (b) % change in *V*_R20_ is plotted as a function of the % emphysema inside V20.

Figure 6 demonstrates the key results from the emphysema sensitivity analysis and further unravels the differences between patients, such as the two illustrated in Fig. 5. Within the 25 patients analysed in this study, the mean % emphysema in the whole lung was found to be 2.1% (± 3.6%, range 0–13%) using the standard clinical threshold of − 950 HU and increased slightly to a mean of 3.4% (± 4.3%, range 0–14.5%) using the volume adjusted emphysema threshold. Inside the V20 volume the amount of emphysema ranged from 0 to 8.5% for a threshold of − 950 HU (mean 0.9 ± 1.9%) and from 0-8.6% (mean 1.3 ± 2.1%) using the adjusted threshold. The plots in Fig. 6 display the impact of the amount of emphysema outside (Fig. 6a) or inside (Fig. 6b) of the V20 isodose volume, spanning across and extending on from the range found in the patient cohort. Results showed that V_R20_ increased with increasing emphysema present outside V20, this increase was greater in the presence of the tumour. This was because with damaged tissue outside of the V20, the ventilation was redistributed into the V20 dose volume. This made the tissue more important functionally and was associated with a greater reduction in patient measured FEV_1_ post-RT. If emphysema was excluded from the V10 isodose volume and only included in lung regions outside of this, there was only a minimal increase in V_R20_ (up to 5% increase when including up to 37.5% emphysema). This suggests that the proximity of the emphysema to the tumour is important in this effect.

The opposite was observed when emphysema was increased within the V20 volume (Fig. 6b). In this case, the ventilation was diverted outside of the V20 volume resulting in a lower model simulated V_R20_. This was again more pronounced in the presence of the tumour. Patients with lower V_R20_ had less loss in lung function post-RT most likely because this tissue was already not well functioning and the removal of it did not reduce the patient’s lung capacity as much; in some cases, lung function was improved after treatment.

## Discussion

In this study, we presented a new approach to simulate ventilation in lung cancer patients pre-RT. Patient-based airway models were created for 25 subjects using CT scans before RT treatment acquired from a UK-based clinical trial. The ventilation distribution was simulated by incorporating patient-specific tumour (size and location) and emphysema distributions. The model predicted V_R20_ (ventilation within the V20 isodose volume) was found to correlate significantly with the change in FEV_1_ 12-months post-RT. It, therefore, has the potential to be used as an *in silico* predictive biomarker for lung toxicity. This relationship was only evident when both the tumour and emphysema effects were included in the model. Simulations showed that the location of the emphysema, in relation to the RT treatment volume (in particular, V20), was important. Patients with more emphysema within the V20 volume had a reduced simulated V_R20_ and had conserved or improvement in lung function post-RT. The reverse was seen in patients with emphysema outside V20 (increased V_R20_ and greater losses in FEV_1_ post-RT). The modelling framework presented here enables a new approach to understand the disease and treatment-related pulmonary function change in RT lung cancer patients. Our goal is to better understand the factors behind the clinical variability in post-RT pulmonary function. This has the potentially important clinical impact to guide RT planning and decision-making in the future. In this study, we focus on late toxicity as it is a permanent side-effect of RT that stabilises around 12-months, while radiation pneumonitis is a transient process.^1^,^34^

Another key finding in this study was an emphasis on the complexity of this system. Several factors contribute to the simulated V_R20_, including the tumour size and location and emphysema amount and location. The tumour location was important due to the gravitational differences in lung function and according to the location with respect to airways (i.e. the size of the airways and the number of downstream airways that were impacted by the tumour). The emphysema location was important in relation to how it altered the ventilation distribution, especially concerning ventilation within the dose-volume (V20 in this case).

To the authors’ knowledge, this is the first study that has incorporated airway obstruction, tissue compliance changes, and emphysema effects to estimate changes in ventilation in lung cancer patients. Simulations were performed to understand the impact of each of these changes in the patient-based models. The impact of the tumour in relation to the stiffening of acinar tissue within the GTV had a relatively small impact. This effect altered the ventilation within the tumour volume only, with very little impact on flows in other areas of the lungs. As the tumour became larger (range from ~ 10 to ~ 325 cm^3^) there was an increasing reduction of ventilation to the ipsilateral lung, up to a maximum of ~ 4%. This flow was redistributed into the contralateral lung due to the assumption of a constant tidal volume within each subject before and after the disease effects were applied. The assumption of a constant tidal volume was made due to an absence of data available describing changes in tidal volume as a function of emphysema and/or a tumour. The model predicted distribution of ventilation is not very sensitive to changes in tidal volume. In addition, the key ventilation value from our modelling is the VR20, which is a normalised flow value (ventilation in V20 / mean ventilation in the whole lung), therefore we do not expect any errors in this assumption to impact on our findings. While there was a clear relationship between tumour size and the reduction of flow, there was also a large amount of variation due to other variables in the models. One variable related to this was the different tumour locations; for example, a patient with a tumour located in the upper lobe had a lower decrease in ventilation due to the tumour. This is because those regions are gravitationally independent and have lower regional ventilation compared to the gravitationally dependent lower lobes. The impact of constricted airways on ventilation distribution was found to be more pronounced compared to the change of tissue compliance. The number of downstream airways affected by the constriction determined how disrupted the ventilation became. For example, central tumours impacted on larger airways with a large number of downstream acini and caused a larger reduction in ventilation in the ipsilateral lung. Inclusion of the emphysema effects into the model had the largest impact, one reason being the more dispersed nature of the emphysema throughout the lung. Regions with emphysema received lower ventilation, this agrees with previous modelling^7^ and image-based measurements.^40^ To compensate for the reduction in ventilation in regions with emphysema, ventilation increased in healthy tissue regions.

The simulated V_R20_ correlates well with both the change in FEV_1_ percent predicted and the change in FEV_1_, but not as strongly with the change in D_LCO_. This is because our model can only evaluate the flow distribution for subjects, but D_LCO_ is a measurement of the efficiency of gas exchange across the alveolar-capillary membrane. A significant correlation may be found between the ventilation-perfusion ratio within V20 and the change in D_LCO_ using both ventilation and perfusion models in the future.

Efforts to investigate the association between baseline density measurements from CT scans and the changes in density post-RT, and further to predict RILD has been performed in previous studies.^10^ Those studies focused on the change in lung density post-RT but did not correlate those findings with lung function. Other studies have been conducted to correlate regional dysfunction and lung function using the measurements from functional imaging (ventilation/perfusion). Fan *et al*.^12^ used single-photon emission computed tomography (SPECT) perfusion data to predict the lung function post-RT (FEV_1_ and D_LCO_) and found that regional perfusion was a significant (*p*-value: 0.005 to 0.080) predictor for the changes in lung function post-RT, but the correlation was weak (*R*^2^: 0.18 to 0.30). Vinogradskiy *et al*. 36 acquired ventilation images calculated using 4DCT data for 96 lung cancer patients and predicted the toxicity post-RT using the ventilation in V20. However, the reported results were not significant at a 0.05 confidence level. Later, Binkley *et al*.^2^ investigated the association between regional ventilation measured from 4DCT scans and lung function post-RT. The correlation between regional ventilation within the 20 Gy isodose volume was found to be significant (< 0.05) with FEV_1_ post-RT. These previous studies showed weaker correlations between the measured variable and post-RT lung function compared to our simulation study with the same significance level.

Our model represents the impact of both the tumour (tissue stiffening and airway constrictions) and tissue density (emphysema) separately on the ventilation distribution without acquiring functional image data. Unlike the 4DCT ventilation image approach, this model can be used to differentiate the low ventilation regions caused by emphysema and occlusion of tumour which could potentially provide more precise guidance for functional avoidance RT planning. For example, the low functional area of emphysema cannot be recovered after RT meaning it could be targeted with a higher dose, while the low ventilation regions caused by airway obstruction, from the tumour, are recoverable and should be avoided during the planning. The results of our model could be used as a tool to tailor the RT plan, such as extend the radiation field to low ventilation regions caused by emphysema and reduce the dose to the relatively high ventilation regions to improve the quality of life for lung cancer patient post-RT.^15^ This type of RT planning has been termed functional lung avoidance RT and is discussed more below.

Modelling provides the advantage of superposing the effects of disease. Several modelling assumptions were made in the current study. First, we assumed a reduced compliance of all acini within the GTV. Lung cancer cells are uncontrolled abnormal cells that cannot retain the same function as normal healthy tissue.^24^ Thus, we believe the assumption that the tumour itself behaves as a non-functional consolidated tissue with higher regional tissue density is valid. Second, airways in the region of the tumour were constricted (between 30 and 50%) during the simulation to represent the occlusion. The constriction values used were based on the assumption that a tumour was unlikely to completely occlude the airways around it. Central airways were constricted to 50% and distal airways to 30% of their original diameters to represent mild to moderate airway obstruction.^11^ Constant constriction values were used due to a lack of information able to be acquired from CT scans.

Another assumption made related to the detection and representation of emphysema. After originally applying a constant standard threshold of -950 HU to identify regions of emphysema, it was evident by looking at the CT scans that many regions with emphysema visible to the naked eye were not being ‘allocated’ using this threshold. The emphysema threshold applied in the model was adjusted using the standard Hounsfield-density equation (Equations 1 and 2) under the assumption of constant lung tissue mass at varying lung volumes. This enabled us to scale the threshold value used to identify emphysema to this cohort of patients for which we observed substantial variation in lung volume during CT acquisition. After altering the threshold value as a function of air volume during imaging we saw that emphysema detection was improved. When including emphysema into the model, we applied the same tissue stiffness for normal and emphysematous tissue but different stretch ratios at FRC (thereby including the impact of emphysema *via* hyperinflation, as presented previously by Burrowes *et al*.).^7^ This meant that the healthy tissue was more compliant than the emphysematous tissue. Emphysema regions present increased local tissue compliance and decreased elastic recoil in reality. So, while this may not be the only mechanism operating in reality, our results showed reduced flow in tissue with emphysema and provided evidence that the model was working as expected. This modelling approach has been applied in two previous studies.^7^,^17^ A study by Kim *et al*.^17^ simulated the ventilation in chronic obstructive pulmonary disease (COPD) patients and compared simulated ventilation with measured ventilation using^12^,^9^Xe MRI and V-SPECT scans, finding good correlations between model and measurements.

Current RT planning constrains the dose to limit severe RILD to 5–10% of the patient population.^9^ Thus, there is potential to treat more patients with RT and/or increase the dose without serious side effects for many patients with consequential therapeutic gain.^13^,^33^ Knowledge of regional baseline lung function pre-RT could be beneficial for treatment planning as well as improving the prediction of patient outcomes post-RT. One such method is functional lung avoidance RT. This has been proposed as a method of reducing toxicity in patients receiving RT for lung cancer by preferentially sparring well-ventilated regions of the lung.^15^ For this approach to be viable, regional functional information is required, most often obtained using SPECT, hyperpolarised gas MRI, or 4D-CT registration methods.^16^ The theory is that regions of existing dysfunction can be preferentially irradiated thereby minimising the loss in lung function. Our computational modelling provides another tool to obtain knowledge of regional lung function before RT treatment. An added advantage of our model is that it can test the impact of different treatment options—such as escalating dose or the dose region—on post-treatment lung function and the ability to quantify potential function that could be recoverable (i.e. functional reduction due to tumour) and function that is not recoverable (emphysema tissue damage). These aspects are outside of the scope of the current work but will form part of our future applications of these models.

The work presented here is an exploratory, proof of concept study. In future work, we aim to include additional patient-based models into our study and further explore multivariate correlations within the patient data. These types of complicated models currently take around 2–3 h to create per patient, including the image processing and model creation/simulation, and require expert users. The longer-term goal is to be able to extract some combination of predictive measures using baseline clinical data (not necessarily needing to build a full computer model for each or any patients) that will improve on the current clinical decision making with respect to the application of thoracic RT. Alongside providing a new potential method for predicting patient lung function post-RT, this work allows us to understand the underlying biophysical mechanisms (i.e. different aspects of tumour and emphysema effects) contributing to patient outcomes which is very difficult to do clinically. The power of this type of modelling is that we can tightly control and methodically vary the numerous factors impacting on patient’s lung function pre- and post-RT treatment. There is still a lot that needs to be improved on and understood in this field. One aspect missing from this work and other work in the field, is the lack of inclusion of a patient’s underlying biology. It may be that, due to underlying genetics, baseline health, environment, diet, individual microbiology and immune system characteristics, patients may inherently respond differently to tissue damage (from RT in this case). These factors may account for substantial variation in outcomes, but this is unknown.

In summary, our study demonstrated a novel computational modelling approach incorporating tissue compliance, emphysema and airway constriction to predict the ventilation distribution in lung cancer patients. Patient-based models were created using CT imaging data from 25 patients with lung cancer. The largest impact on ventilation was the impact of emphysema which affected the acinar compliance. Increasing model-based ventilation heterogeneity showed a statistically significant correlation with patient values of FEV_1_ % predicted at baseline. The model predicted ventilation in the V20 dose-volume correlates with the change in FEV_1_ 12-months post-RT; patients with lower ventilation within V20 tended to have an improved FEV_1_ post-RT. These correlations compare well with previous clinical studies providing some validation that our model is realistically predicting ventilation for these lung cancer patients.

## Electronic supplementary material

Below is the link to the electronic supplementary material.

Electronic supplementary material 1 (DOCX 5034 kb)
